# Ballistic dynamics of flexural thermal movements in a nanomembrane revealed with subatomic resolution

**DOI:** 10.1126/sciadv.abn8007

**Published:** 2022-08-19

**Authors:** Tongjun Liu, Jun-Yu Ou, Nikitas Papasimakis, Kevin F. MacDonald, Vitalyi E. Gusev, Nikolay I. Zheludev

**Affiliations:** ^1^ Optoelectronics Research Centre and Centre for Photonic Metamaterials, University of Southampton Highfield, Southampton SO17 1BJ, UK.; ^2^ Laboratoire d’Acoustique de l’Université du Mans (LAUM), Institut d’Acoustique-Graduate School (IA-GS), CNRS, Le Mans Université, 72085 Le Mans, France.; ^3^ Centre for Disruptive Photonic Technologies and The Photonics Institute, SPMS, Nanyang Technological University, 637371, Singapore.

## Abstract

Flexural oscillations of freestanding films, nanomembranes, and nanowires are attracting growing attention for their importance to the fundamental physical and optical properties and device applications of two-dimensional and nanostructured (meta)materials. Here, we report on the observation of short–time scale ballistic motion in the flexural mode of a nanomembrane cantilever, driven by thermal fluctuation of flexural phonons, including measurements of ballistic velocities and displacements performed with subatomic resolution, using a free electron edge-scattering technique. Within intervals <10 μs, the membrane moves ballistically at a constant velocity, typically ~300 μm/s, while Brownian-like dynamics emerge for longer observation periods. Access to the ballistic regime provides verification of the equipartition theorem and Maxwell-Boltzmann statistics for flexural modes and can be used in fast thermometry and mass sensing during atomic absorption/desorption processes on the membrane.

## Teaser

Phonon-driven thermal flexural motion of a nanomembrane is a sequence of nanometric movements with constant velocities.

## INTRODUCTION

Flexural deformations and modes of oscillation are now understood to be of fundamental importance to the thermal, optical, electrical, and mechanical properties of graphene and other two-dimensional (2D) materials (*1*–*5*) and to the optical properties of photonic metamaterials through near-field coupling among resonators and mechanochromic effects (*6*, *7*). In contrast to a classical Brownian particle in a fluid that is thermally perturbed by external collisions with ambient atoms, thermal movements under vacuum are driven internally by momentum transfer from the annihilation and creation of the flexural phonons.

As long ago as 1906, Einstein realized that the commonly held picture of diffusive thermal motion, characterized by erratic, discontinuous changes in speed and direction, must break down at short time and length scales, where inertia becomes substantial (*8*)—objects must move ballistically between “collision” events. He concluded that this regime of motion would be impossible to observe as to do so would require, at the time, unimaginably high spatial and temporal measurement resolution. Even today, it is a challenging proposition: While such measurements have been reported in recent years for optically trapped microparticles undergoing Brownian motion in gas (*9*) and liquid (*10*), the phonon-dominated dynamics of freestanding films, nanomembranes, nanowires, and cantilevers remain underexplored because there have been no routinely available technologies for quantifying their short–time scale nano/picoscale motion.

We show here that detection of variations in secondary electron emission from the edge of a moving (oscillating) nanomembrane interrogated with a focused electron beam provides for measurements of the membrane’s position with microsecond temporal resolution and subatomic displacement sensitivity. The detection method reveals the Einstein-predicted ballistic regime of thermal flexural motion of the membrane in short time scales. Our experiments allow the measurement of velocities of consecutive steps of membrane movement and their statistics and provide direct experimental verification of the applicability of the equipartition theorem and Maxwell-Boltzmann statistics to flexural dynamics.

## RESULTS

**Fig. 1.**
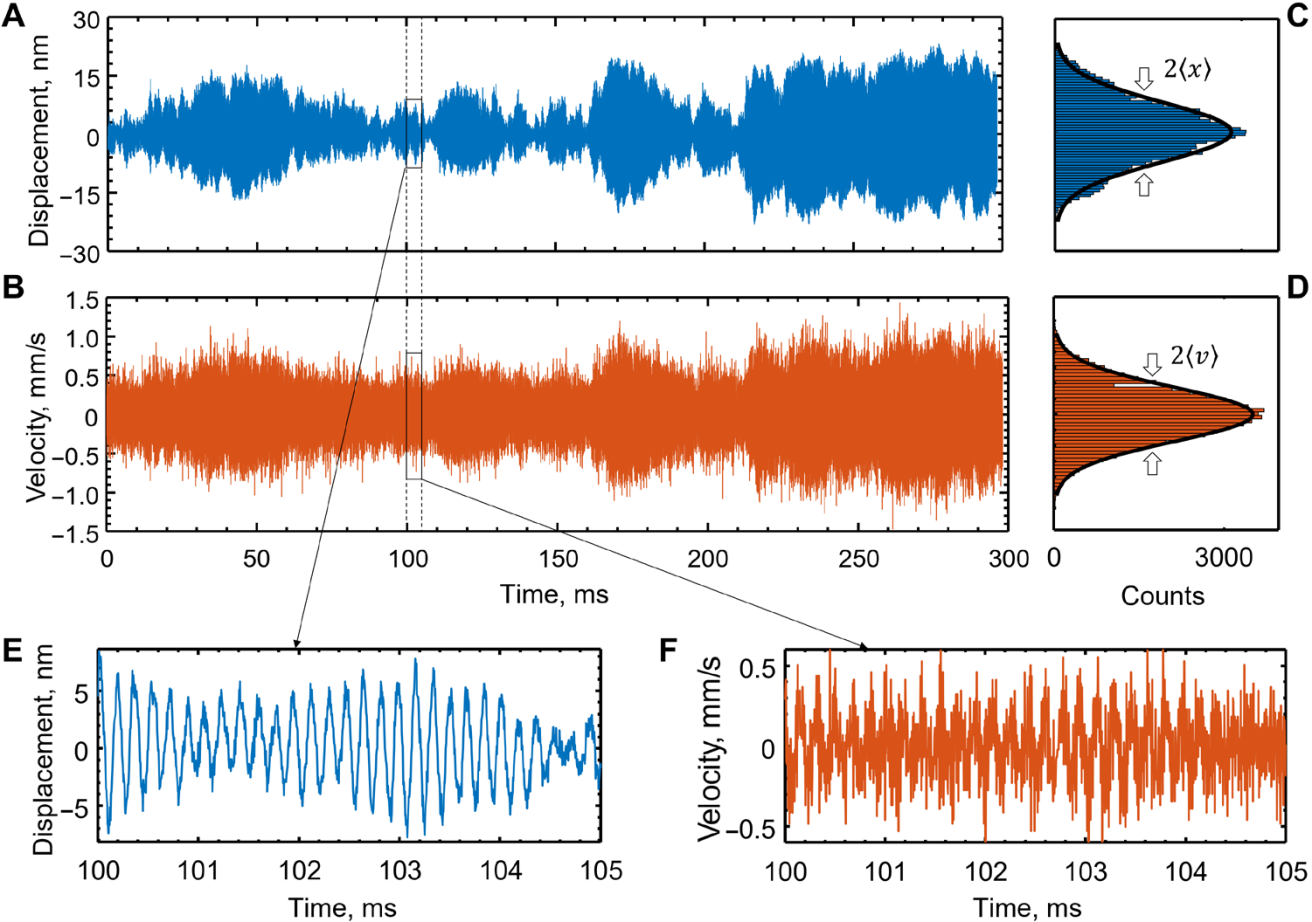
Thermomechanical motion of a gold nanomembrane cantilever measured by free electron edge scattering. Time series recording of displacement (**A**) and corresponding (derived) velocity (**B**) of the tip of a cantilever moving in its fundamental flexural mode under vacuum. (**C**) and (**D**) show corresponding displacement and velocity distributions. Overlaid black lines are Gaussian fittings. (**E**) and (**F**) show zoomed-in sections of (A) and (B), respectively, in which the oscillatory period of the mode θ = 1.75 × 10^−4^ s is resolved.

We investigated the dynamics of thermal motion in the out-of-plane flexural mode of a cantilever cut from a freestanding gold nanomembrane. The material and geometry were selected to facilitate observation of the ballistic regime by consideration of the cantilever’s effective mass, the natural frequency and quality factor of its fundamental flexural mode, and the secondary electron yield. The cantilever was 30 nm thick and 62 μm long with a width tapered from 0.6 μm at the fixed end to 3 μm at the other, and an effective mass *m*_eff_ = 47 pg (see the Supplementary Materials). The secondary electron flux generated by scattering of an electron beam tightly focused on the edge of the membrane is highly sensitive to its position (see Materials and Methods). Position measurements as a function of time reveal Gaussian distributions of the membrane’s position and velocity with root-mean-square values of 〈*x*〉 = 8.3 nm and 〈*v*〉 = 0.30 mm/s, respectively (Fig. 1). The natural oscillation frequency of the cantilever ω_0_/2π = 5.7 kHz and the damping time τ*_b_* = 1/γ = 14 ms were evaluated from the Fourier spectrum of the displacement autocorrelation function.

**Fig. 2.**
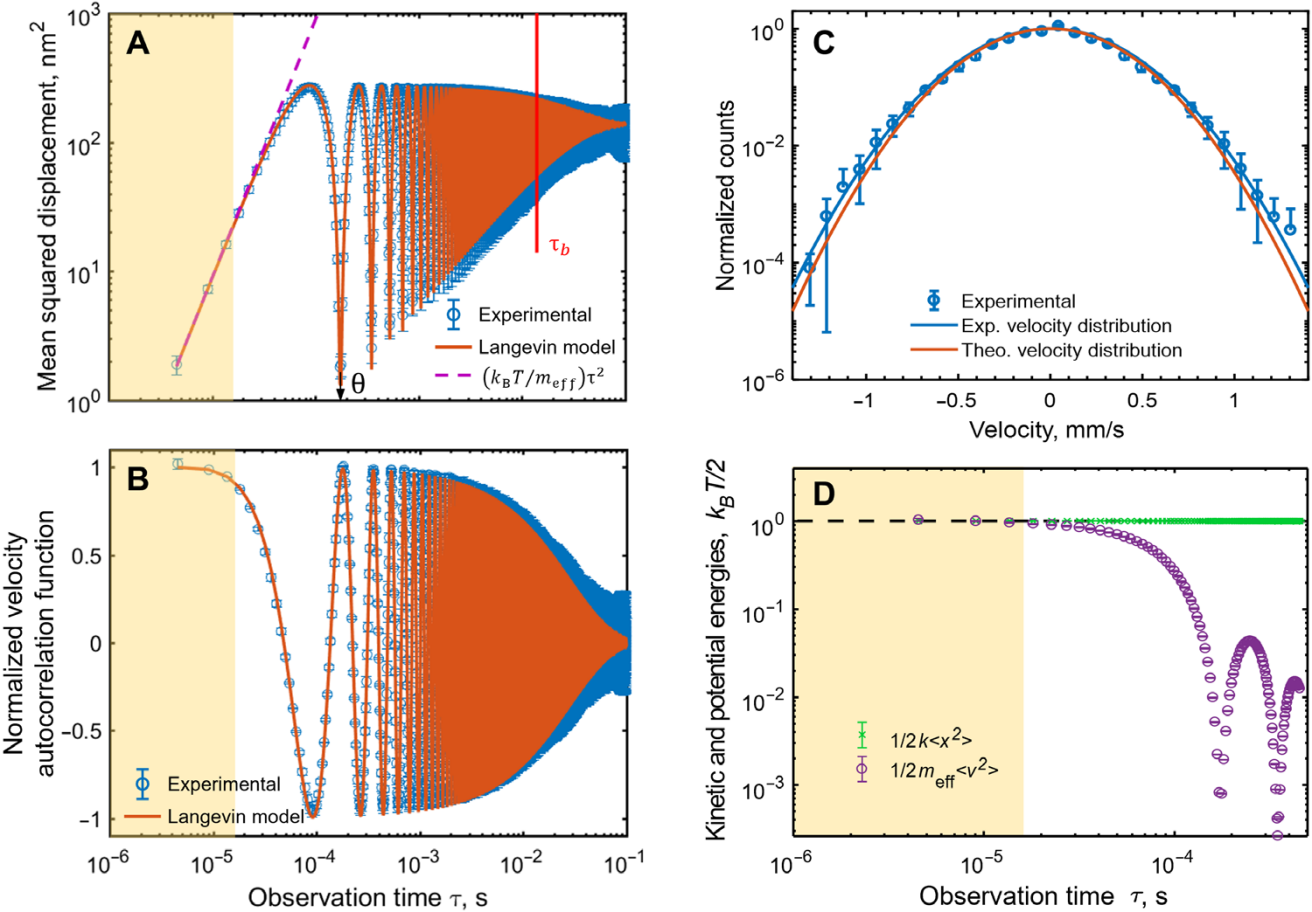
Statistics of nanomembrane cantilever thermal motion—Comparison between experiment and analytical model. (**A**) Mean squared displacement 〈δ*x*(τ)^2^〉 of the membrane cantilever tip as a function of the observation time interval τ. Experimental data are plotted as blue circles. The orange line is derived from the Langevin model for thermal motion of a harmonic oscillator. The violet dashed line is an asymptote for ballistic motion at a constant velocity of $\sqrt{k_{B}T/m_{\text{eff}}}$ = 0.297 mm/s. (**B**) Normalized velocity autocorrelation function as a function of the observation time interval, again, showing experimental data (blue points) overlaid with analytical theory (orange line). (**C**) Measured cantilever tip velocity distribution for an observation time interval τ = 4.7 μs (blue circles). Solid lines are Maxwell-Boltzmann distributions: blue, as a best fit to the experimental data, with 〈*v*〉 = 0.303 mm/s; orange, with 〈*v*〉 = 0.297 mm/s from equipartition theorem. (**D**) Experimentally measured values of $\frac{1}{2}k\langle x^{2}\rangle$ (potential energy, green symbols) and $\frac{1}{2}m_{\text{eff}}\langle v^{2}\rangle$ (kinetic energy, purple line) as functions of the observation time τ. Error bars on experimental data points represent the standard deviation over a number of repeated independent measurements. Yellow shaded zones in (A), (B), and (D) denote the ballistic regime.

To reveal the detailed nature of nanomembrane cantilever thermal motion, from the experimental data, we evaluate the mean squared displacement 〈δ*x*(τ)^2^〉 as a function of observation time τ (Fig. 2A). For small observation intervals τ ≪ θ (where θ = 1.75 × 10^−4^ s is the oscillation period of the cantilever), the mean squared displacement 〈δ*x*(τ)^2^〉 grows quadratically with τ. This is direct evidence of the ballistic motion regime, as it means that velocity is constant over the observation interval. This is confirmed by the normalized velocity autocorrelation function 〈*v*(*t*)*v*(*t* + τ)〉/(*k*_B_*T*/*m*_eff_) plotted in Fig. 2B, which evaluates how close the velocity at the end of the observation period is to the velocity at the beginning. At short time intervals, the near-unity value of the normalized autocorrelation function is again explicit evidence of the ballistic regime. From Fig. 2A, one can conclude that over intervals up to ~10^−5^ s, the cantilever essentially moves ballistically over average distances up to 3 nm.

In essence, the ballistic regime implies that within a short observation interval τ ≪ θ, the natural oscillation of the cantilever is not significantly disturbed by momentum transfer related to the annihilation and creation of individual flexural phonons. The emission and absorption of thermal photons makes a negligible contribution because of their low momentum. At room temperature (*T* = 300 K), the average number of thermal phonons (*11*, *12*) with energy ℏω_0_ in the flexural mode is ${\bar{n}}_{\text{thermal}}\approx k_{B}T/\hbar \omega$_0_ = 1.1 × 10^9^, while the average lifetime of the flexural phonons can be evaluated as ${\left({\bar{n}}_{\text{thermal}}\gamma \right)}^{-1}$ = 13 ps, during which time the cantilever moves an average distance of $\langle v\rangle /({\bar{n}}_{\text{thermal}}\gamma )$ = 3.9 fm. We observe that phonon momentum transfer events begin to affect the ballistic regime of natural oscillation only when the observation intervals τ > 10^−5^ s, i.e., about 6% of the oscillation period θ. During this period of observation, ${\bar{n}}_{\text{thermal}}\gamma \tau$ ~10^6^ phonons of the ~1.1 × 10^9^ in the mode are created and dissipated.

For longer observation periods, 〈δ*x*(τ)^2^〉 becomes a complex oscillating function of τ. A truly diffusive regime, wherein 〈δ*x*(τ)^2^〉 ∝ τ and which is characteristic of free Brownian particle movement, is not observable in cantilevers, where the dynamics are affected by a restoring force. The statistical properties of cantilever thermal motion are described by the Langevin model (*13*, *14*) for a harmonic oscillator (see the Supplementary Materials): $\ddot{x}+\gamma \overset{\cdot }{x}+\omega_{0}^{2}x=F_{\text{thermal}}(t)/m_{\text{eff}},$ where $F_{\text{thermal}}(t)=\sqrt{2k_{B}T\gamma m_{\text{eff}}}\eta (t)$ is the thermal force related to the dissipation factor γ through the fluctuation-dissipation theorem (*15*), *k*_B_ is the Boltzmann constant, and η(*t*) is a delta-correlated normalized white noise term: 〈η(*t*)〉 = 0; 〈η(*t*)η(*t*′)〉 = δ(*t* − *t*′).There is an exceptionally good correlation between experimental data (blue data points in Fig. 2, A and B) and the Langevin model predictions (orange lines) over the entire range of observation times, particularly at τ *< <*θ, where the value of 〈δ*x*(τ)^2^〉 accurately follows the τ^2^*k*_B_*T*/*m*_eff_ dependence derived from the model. The analytical curves are not fittings to the data using any kind of scaling parameters—the near-perfect match seen in Fig. 2 is obtained using only the measured values of *m*_eff_, ω_0_, and γ.

In the ballistic regime of motion (τ ≪ θ), nanomembrane cantilever velocity *v* is well defined, so the distribution of velocities over an ensemble of sampling events can be established, as plotted in Fig. 2C for the shortest observation interval τ = 4.7 μs. A Maxwell-Boltzmann distribution fitting to the data yields a root-mean-square velocity 〈*v*〉 = 0.303 mm/s that is well matched to the value obtained from the energy equipartition theorem: $\sqrt{k_{B}T/m_{\text{eff}}}$ = 0.297 mm/s. The small 2% discrepancy between these values is related to the shot noise of the electron beam current in the experimental case. Compliance with the equipartition theorem, which stipulates that the Boltzmann energy *k*_B_*T* should be equally distributed between potential and kinetic energies of the cantilever, is also illustrated in Fig. 2D. Here, we plot experimental values of $\frac{1}{2}k\langle x^{2}\rangle$ and $\frac{1}{2}m_{\text{eff}}\langle v^{2}\rangle$ as functions of the observation time interval τ (*k* being the cantilever’s spring constant - see Supplementary Materials). The former equates to the potential energy of the cantilever; and the latter equates to kinetic energy only at short intervals τ ≪ θ, where *v* represents the well-defined ballistic velocity. Convergence of the two traces at short time scales thereby confirms equipartition of kinetic and potential energies in the ballistic regime (the yellow shaded band in Fig. 2D).

## DISCUSSION

Our observations of the thermomechanical motion of a nanomembrane cantilever reveal the following dynamics: At short time scales, up to about 10 μs, the membrane moves with constant velocity (i.e., ballistically), with the average displacement being directly proportional to the observation time interval. Brownian-like dynamics emerge for longer observation times, when membrane motion is caused by multiple phononic creation/annihilation events: Average displacement becomes a sub-linear function of the observation time. When the length of the observation interval becomes equal to the natural oscillation period, average displacement reaches a minimum. For intervals much longer than the oscillation period, the mean squared displacement $2k_{B}T/(m_{\text{eff}}\omega_{0}^{2})$ is proportional to temperature and is independent of observation time. High sampling rate measurements of the instantaneous trajectory of the cantilever provide direct verification of thermal equipartition theorem and the Maxwell-Boltzmann distribution of velocities for the membrane.

The secondary electron edge-scattering methodology for detection of nano- to picoscale motion can be deployed on conventional scanning electron microscopes to interrogate thermal (Brownian), self-propelled, and externally driven motion. With spatial resolution (of electron microscopy imaging) that is far beyond the diffraction limit applicable to optical vibrometry techniques, it enables highly selective interrogation and mapping of a target object’s oscillatory modes. It can be applied to measuring subatomic displacements for the study of Van Der Waals and optically induced forces, micro/quantum gravity in nanosystems and materials/device characterization.

The ballistic regime found at short time intervals may be exploited in nanomechanical devices where knowledge of well-defined velocities and of the positions of functional nanocomponents can give advantage. Micro-/nanocantilevers are, for example, widely used as sensor elements (*16*) in physical, chemical, and biological sciences, with measurements based on oscillator characteristics evaluated at thermal equilibrium, on long time scales. Short-interval measurements in the ballistic regime present opportunities for fast thermometry, based on evaluation of the initial *k*_B_*T*/*m*_eff_ slope of mean squared displacement on the observation interval. In being based only on the fundamental rules of thermodynamics and knowledge of the material and geometrical parameters of the cantilever, the calibration of such a thermometry would not require reference to any external standard. The slope measurement may also be used for fast monitoring of cantilever mass at known temperature, for instance, during materials deposition processes or for the detection of molecular adsorption and desorption. From a fundamental perspective, the ability to measure the instantaneous velocity of micro-/nanomechanical structures will be of importance in the study of nonequilibrium statistical mechanics, for instance, in exploring non-Markovian processes and the departure from equipartition theorem expected at the quantum ground state of a cantilever with nonvanishing kinetic energy at 0 K. It may also be important to account for the ballistic nature of motion at short time scales in understanding the electrical, thermal, and mechanical properties of 2D materials (*1*–*4*, *17*).

## MATERIALS AND METHODS

Measurements of picometric (subatomic scale) cantilever displacement are performed by monitoring the secondary electron current generated by scattering of a tightly focused electron beam on a sharp edge on the cantilever: This technique relies upon the fact that the secondary electron current *I* is sensitive to small movements δ*x*(*t*) of the object, giving rise to changes in the current proportional to its gradient in the displacement direction: δ*I*(*t*) ∼ ∇*I* × δ*x*(*t*). The current and its gradient can be measured in the conventional mode of (static) secondary electron imaging. Fluctuations in cantilever position δ*x*(*t*) can then be measured from variations in current δ*I*(*t*).

Here, we used the beam of a scanning electron microscope, with an acceleration voltage of 5 kV and a beam current of 690 pA. For high sensitivity to thermal motion of the cantilever in its fundamental out-of-plane flexural mode (at room temperature under vacuum at 2.6 × 10^−6^ mbar), the electron beam was positioned at the cantilever tip (as indicated in fig. S2A), with the sample plane inclined at 45° to the incident beam direction. Under these conditions, the effects of electron beam–induced heating and momentum transfer to the cantilever are negligible (see the Supplementary Materials).

The noise equivalent displacement sensitivity of the technique, which is determined by the sharpness of the membrane edge, size of the focused electron beam spot, and shot noise of the secondary electron current, reaches ~1 pm/Hz^1/2^.

## Supplementary Materials

### This PDF file includes:

Supplementary Text Figs. S1 and S2 References
