# Supplementary material for: Ballistic dynamics of flexural thermal movements in a nanomembrane revealed with subatomic resolution
Source: Sci Adv. 2022 Aug 19;8(33):eabn8007. doi: 10.1126/sciadv.abn8007 (PMC9390981; doi:10.1126/sciadv.abn8007)
Supplement: Supplementary file 1 — Supplementary Text Figs. S1 and S2 References [file sciadv.abn8007_sm.pdf]

Science Advances
AAAS

Supplementary Materials for

Ballistic dynamics of flexural thermal movements in a nanomembrane revealed with subatomic resolution

Tongjun Liu *et al.*

Corresponding author: Jun-Yu Ou, bruce.ou@soton.ac.uk; Kevin F. MacDonald, kfm@orc.soton.ac.uk

Sci. Adv. **8**, eabn8007 (2022)
DOI: 10.1126/sciadv.abn8007

This PDF file includes:

Supplementary Text
Figs. S1 and S2
References

Supplementary Text

Micro-cantilever geometry, fabrication, effective mass and mechanical quality factor

The cantilever employed in this study was 30 nm thick and 62 μm long, with a trapezoidal shape - 3 μm wide at the unclamped end and 0.6 μm wide at the clamped end (Fig. S1). It was manufactured in a freestanding gold film by focused ion beam milling. The gold film was prepared by evaporation of 30 nm gold onto a silicon nitride membrane, with the silicon nitride then removed by reactive ion etching.

For thermomechanical displacement measurements the incident electron beam is positioned at the center of the short edge of the cantilever tip, as indicated in Fig. S2a. Figure S2b shows the power spectral density (PSD) displacement calculated from measured displacement time-series data, clearly revealing thermal motion at the cantilever's fundamental resonance frequency against a noise floor set by secondary electron detection shot noise.

The effective mass m_{eff} and mechanical quality factor Q for the cantilever's fundamental oscillatory mode are obtained by fitting the following analytical expression for displacement power spectral density $S(f)$, from Wiener–Khinchin theorem (*12, 18*) to the experimental data:

$$S(f) = \frac{k_B T f_0}{2\pi^3 m_{\mathrm{eff}} Q[(f_0^2 - f^2)^2 + (f f_0/Q)^2]} \tag{s1}$$

This fitting – the orange curve in Fig. S2b – yields values m_{eff} = 47.0 ± 0.1 pg (~$^2/_3$ the cantilever's estimated 72 pg real mass, from dimensions and the density of gold), and Q = 501.

Uncertainty in evaluating effective mass is related ultimately to uncertainty in determination of the secondary electron gradient at the measurement point. It is quantified as the standard deviation over several independent measurements of power spectral density at (nominally) the same point on the cantilever. It translates to a relative error of 7.5×10^{-4} in the measurements of RMS velocity.

Disturbance of cantilever movement by the probe electron-beam

The increase in cantilever temperature induced by injection of probe electrons has been evaluated via numerical modelling. Monte Carlo simulations (*19*) show that >95% of 5keV electrons are stopped within the 30 nm thickness of the beam. In keeping with prior works (*20*) we assume that around 2.5% of incident electron beam power is absorbed as heat $H = 0.025IV$ ~100 nW. Taking the specific heat capacity of gold to be 700 $\mathrm{Jkg^{-1}K}$, the cantilever temperature is increased by electron bombardment by δT ~ 0.4 K resulting in a negligible relative increase of the root mean square thermal displacement of the cantilever by only one part in $(\sqrt{1 + \delta T/T} - 1)$ ~7×10^{-4}.

The force related to momentum transfer from the electron beam is $F = \frac{I}{e}\sqrt{2Em_e}$ ~1.6×10^{-13} N. Assuming a spring constant for the cantilever of 60 μN/m (from finite element modelling), this is sufficient to induce static tip displacement of ~2.6 nm. This is much smaller than the (30 nm) thickness of the cantilever, equating to cantilever rotation about the anchor point of only ~0.002°, and is of no consequence to its thermal motion dynamics.

The Langevin model

The Langevin equation for the thermal motion of a damped harmonic oscillator in one dimension is given by Newton's second law with a thermal driving force:

$$\ddot{x}(t) + \gamma\dot{x}(t) + \omega_0^2 x(t) = F_{thermal}(t)/m_{eff} \tag{s2}$$

wherein said force

$$F_{thermal}(t) = \sqrt{2k_B T\gamma m_{eff}}\,\eta(t) \tag{s3}$$

Here, $x(t)$ is the coordinate of the oscillator as a function of time t, m_{eff} is the effective mass, γ is the dissipation factor, $\omega_0 = \sqrt{(k/m_{eff})}$ is the natural angular frequency of oscillation, k is Hooke's spring constant. $F_{thermal}(t)$ is related to dissipation factor γ and ambient temperature T through fluctuation-dissipation theorem.

The dissipation and attenuation of energy in a mechanical structure is thermodynamically required: a mechanical normal mode achieves thermal equilibrium with its environment. The amplitude of this thermal noise is explicitly expressed in terms of dissipation factor γ, the Boltzmann energy $k_B T$, and $\eta(t)$, which is a normalized white noise

$$\langle\eta(t)\rangle = 0;\ \langle\eta(t)\eta(t')\rangle = \delta(t-t') \text{ for all } t, t' \tag{s4}$$

By transforming the Langevin equations to the frequency domain:

$$-\omega^2 x(\omega) - i\omega\gamma x(\omega) + \omega_0^2 x(\omega) = F_{thermal}(\omega)/m_{eff} \tag{s5}$$

one can solve for mechanical susceptibility $x(\omega)$ in terms of the fluctuating force, to obtain

$$x(\omega) = \frac{F_{thermal}(\omega)}{m_{eff}}\frac{1}{\omega_0^2-\omega^2-i\gamma\omega} \tag{s6}$$

where $F_{thermal}(\omega) = \sqrt{2k_B T\gamma m_{eff}}$. The Wiener-Khinchin theorem states that the power spectral density of a wide-sense stationary random process, i.e., a stochastic process with a constant mean (here $x(t)$), is equal to the Fourier transform of its autocorrelation function, and vice versa:

$$S_{xx}(\omega) = \int_{-\infty}^{+\infty} d\tau e^{-i\omega\tau}\langle x(t)x^*(t+\tau)\rangle \tag{s7}$$

The doubled-sided (containing both negative and positive frequency components) displacement power spectral density (PSD) $S_{xx}(\omega) = \langle x(\omega)x^*(\omega)\rangle$ is proportional to $|x(\omega)|^2$, so

$$S_{xx}(\omega) = \frac{2k_BT\gamma}{m_{eff}} \frac{1}{(\omega_0^2-\omega^2)^2+\gamma^2\omega^2} \tag{s8a}$$

$$S_{vv}(\omega) = \frac{2k_BT\gamma}{m_{eff}} \frac{\omega^2}{(\omega_0^2-\omega^2)^2+\gamma^2\omega^2} \tag{s8b}$$

Therefore, the single-sided (containing only positive frequency components) thermomechanical displacement power spectral density (PSD) of a nanomechanical resonator (i.e., $S_x = 2S_{xx}$), in terms of its effective mass m_{eff}, quality factor $Q = \omega_0/\gamma$ and resonant frequency $f_0 = \omega_0/2\pi$, is

$$S_x(\omega) = \frac{4k_BT\gamma}{m_{eff}} \frac{1}{(\omega_0^2-\omega^2)^2+\gamma^2\omega^2} \tag{s9}$$

which is essentially equation (s1) in the angular frequency domain.

Position autocorrelation is the inverse Fourier transform of displacement power spectral density, according to Wiener-Khinchin theorem. Therefore,

$$\langle x(t)x(t+\tau)\rangle = \frac{1}{2\pi}\int_{-\infty}^{+\infty} S_{xx}(\omega)e^{i\omega\tau}d\omega = \frac{1}{2\pi}\int_{-\infty}^{+\infty} \frac{2k_BT\gamma}{m_{eff}} \frac{1}{(\omega_0^2-\omega^2)^2+\gamma^2\omega^2} d\omega \tag{s10}$$

Above integration kernel has four singularities, when $(\omega_0^2-\omega^2)^2 + \gamma^2\omega^2 = 0$, with

$$\omega = \pm\frac{i\gamma}{2} \pm \sqrt{\omega_0^2 - (\gamma/2)^2} \tag{s11}$$

The damped angular frequency defined as

$$\omega_1 = \sqrt{\omega_0^2 - (\gamma/2)^2} \tag{s12}$$

Depending upon the value of ω_1, one can identify three different regimes: $\omega_1 > 0$ corresponds to an underdamped oscillator; $\omega_1 = 0$ a critically damped oscillator; and ω_1 complex valued an overdamped oscillator. The integral in equation (s10) can be calculated as a contour integral in the complex plane of ω by taking the residues at the poles located at values of equation (s11), while only considering the upper half semi-circle as observation time interval $\tau > 0$.

For under/over damped cases, one obtains a position autocorrelation function:

$$\langle x(t)x(t+\tau)\rangle = \frac{k_BT}{m_{eff}\omega_0^2} e^{-\frac{\tau}{2\tau_b}} \left(cos\omega_1\tau + \frac{sin\omega_1\tau}{2\omega_1\tau_b}\right) \tag{s13}$$

with momentum relaxation time $\tau_b = 1/\gamma$. Note that at $\tau = 0$, $\langle x(t)^2\rangle = k_BT/m_{eff}\omega_0^2$ - corresponding simply to equipartition theorem $1/2\, m_{eff}\omega_0^2\langle x(t)^2\rangle = 1/2k_BT$.

The mean squared displacement (MSD) of a Brownian underdamped harmonic oscillator - a measure of average distance travelled at a given time interval τ (**the orange line in Fig. 2a**), can thus be obtained through $\langle|x(t)-x(t+\tau)|^2\rangle = 2\langle x^2(t)\rangle - 2\langle x(t)x(t+\tau)\rangle$ as:

$$MSD(\tau) = \langle|x(t)-x(t+\tau)|^2\rangle = \frac{2k_BT}{m_{eff}\omega_0^2}\left[1-e^{-\frac{\tau}{2\tau_b}}\left(cos\omega_1\tau + \frac{sin\omega_1\tau}{2\omega_1\tau_b}\right)\right] \tag{s14}$$

For the critically damped case, the position correlation function reads

$$\langle x(t)x(t+\tau)\rangle = \frac{k_BT}{m_{eff}\omega_0^2}e^{-\frac{\tau}{2\tau_b}}(1+\tau/\tau_b) \tag{s15}$$

And mean square displacement is

$$MSD(\tau) = \langle|x(t)-x(t+\tau)|^2\rangle = \frac{2k_BT}{m_{eff}\omega_0^2}\left[1-\left(1+\frac{\tau}{2\tau_b}\right)e^{-\frac{\tau}{2\tau_b}}\right] \tag{s16}$$

For short time scales $\tau \ll \tau_b$ or $\tau \to 0$, by using the Taylor series $e^{-t} = 1 - t + t^2/2! + O(t^3)$, $\sin(t) = t + O(t^3)$ and $\cos(t) = 1 - t^2/2! + O(t^4)$, one obtains respectively from equations (s14) and (s16),

$$MSD(\tau) = \frac{2k_BT}{m_{eff}\omega_0^2}\left[1-\left(1-\frac{\tau}{2\tau_b}+\frac{\tau^2}{8\tau_b^2}+O(\tau^3)\right)\left(1+\frac{\tau}{2\tau_b}-\frac{\omega_1^2\tau^2}{2}+O(\tau^3)\right)\right] \tag{s17}$$

$$MSD(\tau) = \frac{2k_BT}{m_{eff}\omega_0^2}\left[1-\left(1+\frac{\tau}{2\tau_b}\right)\left(1-\frac{\tau}{2\tau_b}+\frac{\tau^2}{8\tau_b^2}+O(\tau^3)\right)\right] \tag{s18}$$

Using equation (s12) and $\tau_b = 1/\gamma$ to simplify these expressions, one obtains the ballistic behavior, respectively, of an under/over damped oscillator with $\omega_1 = \sqrt{\omega_0^2 - 1/(2\tau_b)^2}$ (**the purple line in Fig. 2a**):

$$MSD^{Ballistic}(\tau) = \frac{2k_BT}{m_{eff}\omega_0^2}\left(\frac{1}{8\tau_b^2}+\omega_1^2\right)\tau^2 = \frac{k_BT}{m_{eff}}\tau^2 \tag{s19}$$

and of a critically damped oscillator with $\omega_1 = 0$, i.e., $\omega_0^2 = 1/(2\tau_b)^2$

$$MSD^{Ballistic}(\tau) = \frac{2k_BT}{m_{eff}\omega_0^2}\frac{\tau^2}{8\tau_b^2} = \frac{k_BT}{m_{eff}}\tau^2 \tag{s20}$$

The velocity autocorrelation function (VACF) can be obtained by similar means:

$$\langle v(t)v(t+\tau)\rangle = \frac{1}{2\pi}\int_{-\infty}^{+\infty} S_{vv}(\omega)e^{i\omega\tau}d\omega \tag{s21}$$

Using contour integration, the VACF for an underdamped Brownian oscillator is then given by:

$$\langle v(t)v(t+\tau)\rangle = \frac{k_B T}{m_{eff}} e^{-\frac{\tau}{2\tau_b}} \left(cos\omega_1\tau - \frac{sin\omega_1\tau}{2\omega_1\tau_b} \right) \tag{s22}$$

The normalized velocity autocorrelation function (NVACF), quantifying similarity between the velocity v after time τ and the initial velocity (**the orange line in Fig. 2b**), is

$$NVACF = \psi(\tau) = e^{-\frac{\tau}{2\tau_b}} \left(\cos\omega_1\tau - \frac{\sin\omega_1\tau}{2\omega_1\tau_b} \right) \tag{s23}$$

The Maxwell Boltzmann distribution for velocity v (**the orange line in Fig. 2c**) is

$$f(v) = \sqrt{\frac{m_{eff}}{2\pi k_B T}} exp\left(-\frac{m_{eff}v^2}{2k_B T} \right) \tag{s24}$$

and then velocity variance $v_{rms} = \sqrt{k_B T/m_{eff}}$.

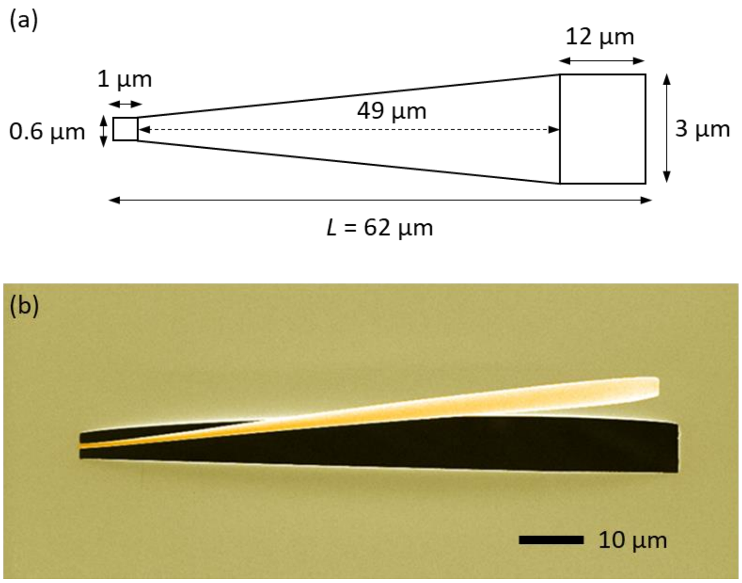

Fig. S1.

Nanomechanical micro-cantilever. (a) In-plane dimensional schematic of the cantilever. (b) False color scanning electron microscope image of the cantilever, taken at a 30° viewing to the free-standing gold membrane surface normal.

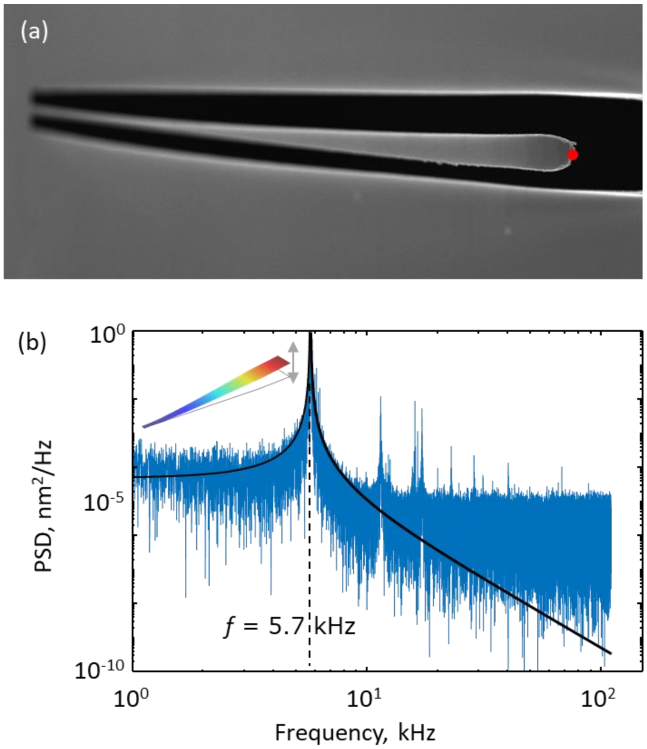

Fig. S2.

Displacement power spectral density at the cantilever tip. (a) Scanning electron microscope image of the cantilever taken in the orientation in which tip-displacement time-series measurements are performed, i.e. with the sample plane inclined at 45° to the incident electron beam [in consequence of which, the left hand side of the image is out of focus]. (b) Displacement power spectral density (PSD) of the cantilever tip measured at the position denoted by the red dot in panel (a). The overlaid black line is a best fit of the analytical expression for PSD given in Eq. S1.

REFERENCES AND NOTES

1. S. V. Morozov, K. S. Novoselov, M. I. Katsnelson, F. Schedin, D. C. Elias, J. A. Jaszczak, A. K. Geim, Giant intrinsic carrier mobilities in graphene and its bilayer. *Phys. Rev. Lett.* **100**, 016602 (2008).

2. E. Mariani, F. von Oppen, Flexural phonons in free-standing graphene. *Phys. Rev. Lett.* **100**, 076801 (2008).

3. A. Taheri, S. Pisana, C. V. Singh, Importance of quadratic dispersion in acoustic flexural phonons for thermal transport of two-dimensional materials. *Phys. Rev. B* **103**, 235426 (2021).

4. L. Lindsay, D. Broido, N. Mingo, Flexural phonons and thermal transport in multilayer graphene and graphite. *Physical Review B* **83**, 235428 (2011).

5. S. Zheng, J. K. So, F. Liu, Z. Liu, N. Zheludev, H. J. Fan, Giant enhancement of cathodoluminescence of monolayer transitional metal dichalcogenides semiconductors. *Nano Lett.* **17**, 6475–6480 (2017).

6. J. Li, D. Papas, T. Liu, J.-Y. Ou, K. F. MacDonald, E. Plum, N. I. Zheludev, Thermal fluctuations of the optical properties of nanomechanical photonic metamaterials. *Adv. Opt. Mater.* **10**, 2101591 (2021).

7. A. Karvounis, N. Aspiotis, I. Zeimpekis, J.-Y. Ou, C.-C. Huang, D. Hewak, N. I. Zheludev, Mechanochromic reconfigurable metasurfaces. *Adv. Sci.* **6**, 1900974 (2019).

8. A. Einstein, Zur Theorie der Brownschen Bewegung. *Ann. Phys. - Berlin* **324**, 371–381 (1906).

9. T. Li, S. Kheifets, D. Medellin, M. G. Raizen, Measurement of the instantaneous velocity of a Brownian particle. *Science* **328**, 1673–1675 (2010).

10. R. Huang, I. Chavez, K.M. Taute, B. Lukić, S. Jeney, M.G. Raizen, E.L. Florin, Direct observation of the full transition from ballistic to diffusive Brownian motion in a liquid. *Nat. Phys.* **7**, 576–580 (2011).

11. K. C. Schwab, M. L. Roukes, Putting mechanics into quantum mechanics. *Phys. Today* **58**, 36–42 (2005).

12. M. Aspelmeyer, T. J. Kippenberg, F. Marquardt, Cavity optomechanics. *Rev. Mod. Phys.* **86**, 1391–1452 (2014).

13. M. C. Wang, G. E. Uhlenbeck, On the theory of the Brownian motion II. *Rev. Mod. Phys.* **17**, 323–342 (1945).

14. S. F. Nørrelykke, H. Flyvbjerg, Harmonic oscillator in heat bath: Exact simulation of time-lapse-recorded data and exact analytical benchmark statistics. *Phys. Rev. E* **83**, 041103 (2011).

15. R. Kubo, M. Toda, N. Hashitsume, *Statistical physics II: Nonequilibrium Statistical Mechanics*. (Springer Science & Business Media, 2012), vol. 31.

16. H. P. Lang, M. Hegner, C. Gerber, in *Springer Handbook of Nanotechnology*. (Springer, 2017), pp. 457–485.

17. L. Shi, Nonresistive heat transport by collective phonon flow. *Science* **364**, 332–333 (2019).

18. B. D. Hauer, C. Doolin, K. S. D. Beach, J. P. Davis, A general procedure for thermomechanical calibration of nano/micro-mechanical resonators. *Ann. Phys. - New York* **339**, 181–207 (2013).

19. D. Drouin, A. R. Couture, D. Joly, X. Tastet, V. Aimez, R. Gauvin, CASINO V2.42—A fast and easy-to-use modeling tool for scanning electron microscopy and microanalysis users. *Scanning* **29**, 92–101 (2007).

20. E. Buks, M. L. Roukes, Stiction, adhesion energy, and the Casimir effect in micromechanical systems. *Phys. Rev. B* **63**, 033402 (2001).
